# A critical look at challenges and future scopes of bioactive compounds and their incorporations in the food, energy, and pharmaceutical sector

**DOI:** 10.1007/s11356-022-19423-4

**Published:** 2022-03-02

**Authors:** Sanidhya Pai, Akshatha Hebbar, Subbalaxmi Selvaraj

**Affiliations:** 1 Department of Biotechnology, Manipal Institute of Technology, Manipal Academy of Higher Education (MAHE), Manipal, 576104 India; 2 Department of Chemical Engineering, Manipal Institute of Technology, Manipal Academy of Higher Education (MAHE), Manipal, 576104 India

**Keywords:** Bioactive compounds, Extraction, Industrial applications, Pharmaceutical, Characterization

## Abstract

Bioactive compounds refer to secondary metabolites extracted from plants, fungi, microbes, or animals. Besides having pharmacological or toxicological effects on organisms leading to utilization in food and pharmaceutical industries, the discovery of novel properties of such compounds has led to the diversification of their applications, ranging from cosmetics and functionalized biomaterials to bioremediation and alternate fuels. Conventional time-consuming and solvent-intensive methods of extraction are increasingly being replaced by green solvents such as ionic liquids, supercritical fluids, and deep eutectic solvents, as well as non-conventional methods of extraction assisted by microwaves, pulse electric fields, enzymes, ultrasound, or pressure. These methods, along with advances in characterization and optimization strategies, have boosted the commercial viability of extraction especially from agrowastes and organic residues, promoting a sustainable circular economy. Further development of microfluidics, optimization models, nanoencapsulation, and metabolic engineering are expected to overcome certain limitations that restrict the growth of this field, in the context of improving screening, extraction, and economy of processes, as well as retaining biodiversity and enhancing the stability and functionality of such compounds. This review is a compilation of the various extraction and characterization methods employed for bioactive compounds and covers major applications in food, pharmacy, chemicals, energy, and bioremediation. Major limitations and scope of improvement are also discussed.

**Graphical abstract:**

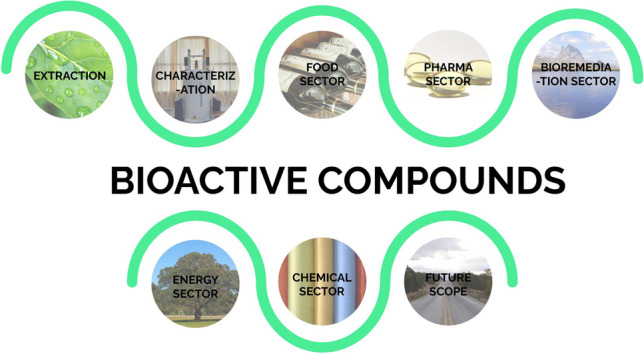

## Introduction

Bioactive compounds engender bioactive properties in the human body without adding any nutritional benefit and fall under secondary metabolites in plants. They express pharmacological or toxicological effects in humans and other animals (Câmara et al. 2020; Azmir et al. 2013). These compounds are procured from foods such as vegetables, fruits, and whole grains. Natural bioactive compounds are classified as polyphenols, triterpenes and phytosterols, terpenoids, polysaccharides, capsaicinoids, carotenoids and tocopherols, alkaloids, saponins glucosinolates, and others (Makkar et al. 2021). They are also extracted from fungi, animals, and bacteria (Câmara et al. 2020), as well as from agro-industrial residues such as avocado peel, mango seeds, and grape peels (Shirahigue and Antonini 2020).

They manifest antioxidant, anti-allergic, anti-inflammatory, antimicrobial, anticarcinogenic, and antimutagenic properties and are essential for the human body. Moreover, they are also vital in the pharmaceutical, food, and chemical industries (Câmara et al. 2020). Bioactive compounds account for numerous health benefits and help to prevent various diseases and metabolic abnormalities which were proved in several pharmacological studies (Chhikara et al. 2019). In the food and fermentation industry, bioactive compounds such as essential oils, flavonoids, tannins, phenolic acids, carotenoids, organosulfur compounds, phytosterols, and tocopherols are used in the processing of vegetable oils, meat and seafood products, bakery products, dairy products, etc. (Shirahigue and Antonini 2020). Bioactive compounds are found to have anti-aging properties which are desirable for the cosmetic industry (Câmara et al. 2020).

Diverse types of extraction strategies have been employed over the years, considering properties such as the nature of source matrix, relative solubility, structure, and chemical properties of both the bioactive compound and solvents used, as well as the effect of temperature, pressure, pH, etc. on the time, yield, and selectivity of extraction (Azmir et al. 2013). Conventional methods of extraction include maceration and decoction, which have been widely used in the extraction of essential oils and other bioactive compounds at a household scale (Muala et al. 2021), as well as the model laboratory-scale Soxhlet extraction method that uses slightly elevated temperatures to recirculate solvent within an apparatus and to aid the extraction of compounds from samples placed in a thimble (Raynie 2019).

However, these methods come with limitations, in terms of high extraction times and bulk solvent use, lower efficiencies when relative yields and specificity of extracted compounds are taken into consideration. For example, maceration requires 2 to 7 days for a satisfactory extraction, and the ratios of solvent to crude extract could vary from 4:1 to even around 50:1 in the case of decoction (hot water extraction) (Li et al. 2020). Often, there are requirements of pure and expensive solvents which are toxic. Elevated temperatures used in some methods are unsuitable for the extraction of thermally unstable compounds (Raynie 2019).

Various methods have been developed to overcome the above limitations, in addition to the development of several new types of solvents that are “green,” less toxic, cost-effective, and more specific (Pal and Jadeja 2020). Ionic liquids, deep eutectic solvents, and aqueous two-phase systems, for instance, have been incorporated to increase yield and speed of extraction, as well as aid in the extraction of compounds that were too difficult to extract with conventional solvents (Priyadarshi et al. 2020). The solvent-bioactive substance interactions could be compared and optimized using several parameters, such as the Hansen parameters and solvation parameter models (Lefebvre et al 2021).

The basic principle of unconventional extraction methods is assisted extraction using ultrasound, pressurized liquids, microwaves, and pulsed electric fields, with the main aim of cell wall rupture or deterioration, thus enhancing mass transfer and facilitating effective mixing due to the exposure of cytoplasmic contents to the solvent (Lefebvre et al. 2021). Ultrasound-assisted extraction (UAE), pressurized liquid extraction (PLE), microwave-assisted extraction (MAE), supercritical fluid extraction (SCFE), and pulse electric field-assisted extraction (PEFAE) are a few unconventional methods following the above principles. Enzyme-assisted extraction (EAE) has also been considered as an option to extract substances associated with the cell wall, rather than the cytoplasm, by employing enzyme-driven cell wall digestion (Azmir et al. 2013). Supercritical fluid extraction is one of the most popularly used extraction strategies, usually employing supercritical CO_2_ due to favorable thermodynamic properties and the renewable nature of the solvent (Gan et al. 2020).

The success and efficiency of any method are also determined based on the choice of solvent; the effect of temperature and pH, which could potentially alter cell wall organization, specificity; chemical structure of the compound to be extracted; and feasibility of the process, among other effects. A suitable combination of parameters is arrived at by implementing optimization strategies specific to the process, as shown by Catarino et al. (2019).

Previous reviews have focused on individual aspects of the extraction of bioactive compounds. Sources, applications, and extraction strategies have been documented separately. We aim to provide a comprehensive review explaining bioactive compounds in the food, chemical, and pharmaceutical industry, their sources, extraction methods, and their limitations, characterization, applications, and future scope.

## Methods of extraction of bioactive compounds

**Fig. 1:**
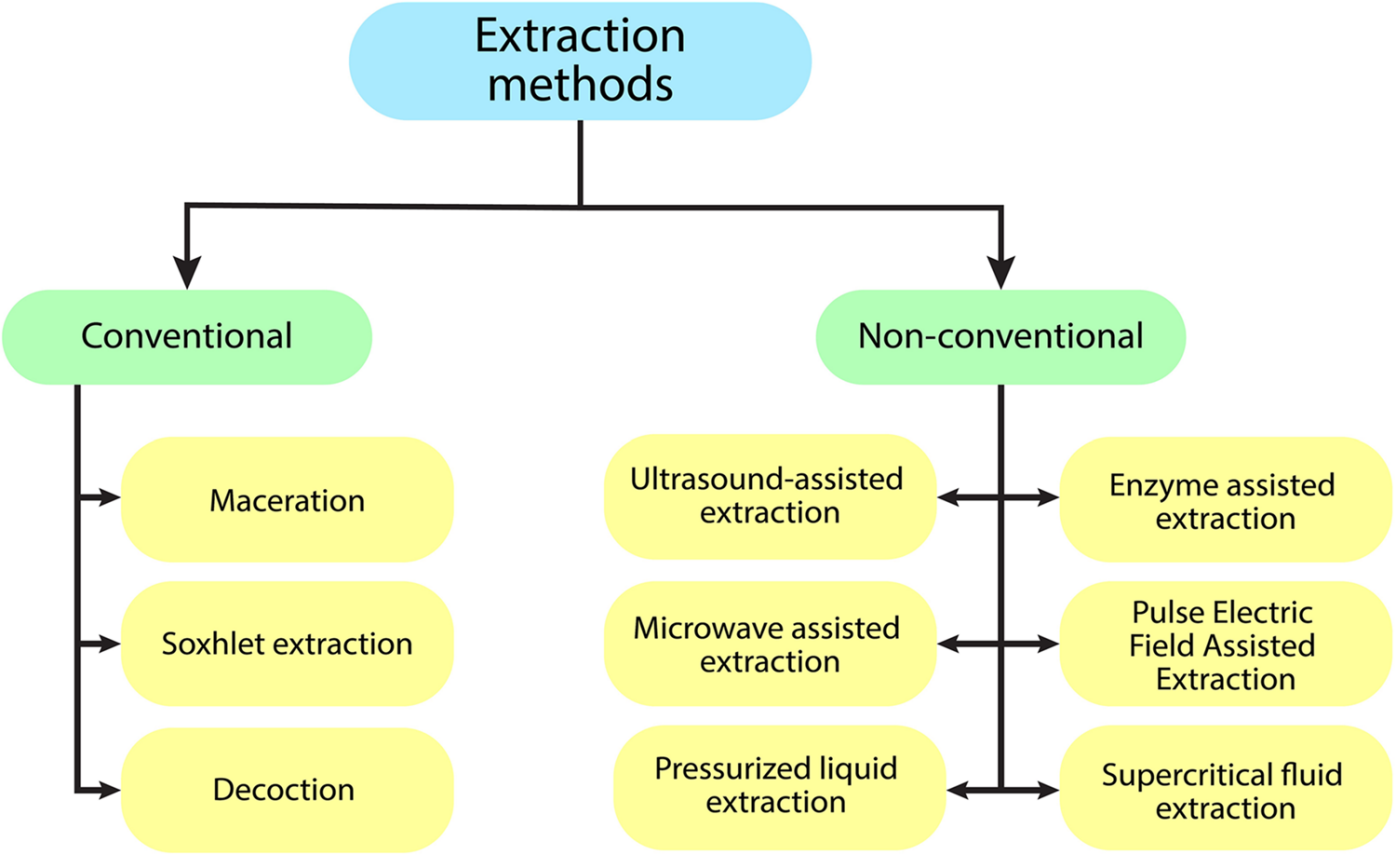
A representation of universally used extraction methods of bioactive compounds

The diversity of primary and secondary metabolites of plants and microbes in nature and their innumerable applications in various fields necessitates the use of a vast array of extraction methods, optimized according to their properties (Zhang et al. 2018). Extraction is the process of obtaining a compound of interest from a raw source. It is broadly classified into two types: conventional and non-conventional. Conventional methods include maceration, decoction, and Soxhlet extraction. Most industrial-scale extraction units rely on solvents (notably, hexane), most of which are products of the petrochemical industry. High energy consumption and bulk use of such solvents have adverse environmental impacts (Pal and Jadeja 2020). Non-conventional methods thus focus on using various physical or enzymatic means to enhance extraction (ultrasound, pressure, pulsed electric fields, microwaves, etc.) while utilizing lesser amounts of solvents or specialized “green” solvents (supercritical fluids, deep eutectic mixtures, etc.) (Anticona et al. 2020). Figure 1 represents the classification of extraction methods, along with specific examples under each category.

### Conventional methods

#### Maceration

Maceration is a table-top extraction method commonly used for the extraction of medicinal plants. Some examples with sources and process conditions are mentioned in Table 1. It involves crushing the raw source coarsely and placing it in a container. The solvent is poured to cover the crushed source completely and is allowed to stand for 3 days with frequent agitation until the soluble matter is dissolved. The mixture is strained and decanted to complete the extraction process (Majekodunmi 2015). Eventually, the extract is separated using evaporation in a water bath. This method is apt for thermolabile plant extracts as it does not require elevated temperatures. However, this extraction process is time-consuming (Zhang et al. 2018).

**Table 1:** Principle involved, along with various sources, compounds, and process conditions employed in maceration

Principle	Sources	Compounds extracted	Process conditions	References
Heating, infusion	Chokeberry fruit	Phenols, Anthocyanins	50% ethanol, 1:20, particle size of 0.75 mm	Zhang et al. 2018
	Dried root of *S. baicalensis* Georgi	Baicalein	70% ethanol	Xie et al. 2019
	*Salvia officinalis*	Rosmarinic acid, Carnosol	Boiling water or 50% hydroethanolic solution, 5 days, room temperature	Vieira et al. 2020
	*Piper betle* (betel) leaves	Eugenol, Eugenol acetate	100% acetone, 1:5, 72 h, room temperature	Das et al. 2019
	*Anthemis cotula L.* (stinking chamomile)	Anthecotuloid, Caffeoyl quinic acid and quercetin	96% ethanol, 1:20, room temperature, overnight	Sut et al. 2019
	Blackcurrant leaves	Polyphenols (TPC), Flavonoids (TFC), and Proanthocyanidin oligomers (OPC)	Water, 1:100, 500 rpm, 7 h, 30 °C	Cao-Ngoc et al. 2020
	Red algae *Gracilaria gracilis*	Allophycocyanins, Phycoerythrins, and Phycocyanins	M phosphate buffer, 10 min; 1:50	Pereira et al. 2020
	Papery skin of *Allium cepa L.* var. *ascalonicum* (fractions of Maja Cipanas onion)	Anthocyanin, Alkaloids, Polyphenols, Tannins, Flavonoids	70% ethanol + HCl (2 N), 1:10 pH: 1, 24 h, 40 °C	Saptarini and Wardati 2020

#### Soxhlet extraction

Soxhlet extraction is a model extraction technique, used to extract compounds, traditionally, lipids, from solid or semi-solid matrices (Talekar et al. 2018). A porous, usually disposable thimble made of cellulose (Raynie 2019) is placed inside an extraction chamber, to hold the sample. The solvent used for extraction is heated in a round-bottomed flask, which is connected to the extraction chamber. Vapor flows into a condenser, and the condensate is directed to the extraction chamber, where extraction occurs. A siphon redirects the solvent along with the extracted compounds back into the flask below. This process is repeated until extraction is complete (Weggler et al. 2020). Table 2 mentions some of the sources and compounds extracted by Soxhlet extraction as well as the corresponding solvents and process conditions involved. This method is used as a benchmark to compare and develop newer methods. It is also relatively easy to automate, without requiring a lot of supervision (Raynie 2019). However, exceptionally long extraction times (12 to 24 h), high energy consumption, and problems in selectivity and efficiency limit the scope of this technique (Weggler et al. 2020; Mussatto 2015; Wianowska and Wiśniewski 2015).

**Table 2:** Principle involved, along with various sources, compounds extracted, solvents used, and the corresponding process conditions used in Soxhlet extraction

Principle	Sources	Compounds extracted	Process conditions	References
Heating, condensation, extraction, and reflux of S within a Soxhlet apparatus	Spent coffee (silverskin)	Caffeine	Hexane, dichloromethane, ethanol, 1:50, 6 h, the temperature was a solvent boiling point	Mussatto et al., 2015
		Chlorogenic acid	60% isopropanol (60%), 1:10, 27 °C	
	Waste *Punica granatum L.* (pomegranate) seeds	Oils (PUFAs, punicic acid)	Hexane, 1:15, 4 h, 60 °C	Talekar et al., 2018
	*Rosmarinus officinalis L.* (rosemary) leaves	Rosmarinic acid, Carnosic acid, Carnosol	96% food grade ethanol, demineralized water, 1:12, 8 h	Hirondart et al. 2020
	*Piper betle* (betel) leaves	Eugenol, Eugenol acetate	100% acetone, 1:5, 56 °C, 8 h	Das et al., 2019
	*Agaricus bisporus L*	Ergosterol	Hexane, ethanol, or limonene (150 mL for 4.5 g of sample), 4 h,	Heleno et al., 2016
	*Anthemis cotula L.* (stinking chamomile)	Anthecotuloid, Caffeoyl Quinic acid, and Quercetin	96% ethanol, 1:20, 6 h	Sut et al, 2019
	*Allium cepa L.* var. *ascalonicum*	Alkaloids, Polyphenols, Tannins, Flavonoids	70% ethanol + HCl (2 N),1:10, pH: 1, 2 h	Saptarini and Wardati, 2020
	*Silybum marianum L. Gaertner* fruits	Silymarin	n-hexane (defatting), methanol (for Silymarin extraction), 2:75, 6 h (defatting) + 5 h (actual extraction)	Wianowska and Wiśniewski, 2015
	*Miscanthus sinensis* (runo) stem	Runo dye	50% ethanol and 4 h	Pinzon et al., 2020

#### Decoction

A decoction is a method of extraction of heat-stable bioactive compounds, obtained by boiling in water, which is usually used as a solvent (Hmidani et al. 2019). It is widely used in traditional medicine in the form of oral formulations containing extracts of certain medicinal herbs, due to its capability of a rapid therapeutic action (Wang et al. 2019). Common sources include hard8 solids such as roots, bark, and seeds, which are ground and heated with water in a closed vessel. The extract is then cooled and filtered from the insoluble residue (Perera et al. 2017). Table 3 gives a general overview of the various compounds extracted by decoction, along with corresponding sources and process conditions. A decoction is characterized by noticeably short extraction times of around 5 to 10 min. However, this might be a disadvantage if the bioactive compounds that must be extracted are not so soluble in water. In addition to this, there is a large solvent to solid ratio involved (Zhang et al. 2018).

**Table 3:** Principle involved, along with the various sources, compounds extracted, and the corresponding process conditions used in decoction

Principle	Sources	Compounds extracted	Process conditions	References
Heating or boiling solids in an aqueous medium	*Syzygium cumini* bark	Gallic acid, Umbelliferone, Ellagic acid	Water, 16 mL/g of powdered bark	Perera et al., 2017
	*Astragalus membranaceus*	Astragaloside IV	Water	Zhang et al., 2020a, b
	Berry (Strawberry, raspberry, blueberry, blackberry)	Anthocyanins, Hydroxycinnamic acids (extractable fraction); Ellagic acid, Hydroxybenzoic acid (hydrolyzable fraction)	Water	Reynoso-Camacho et al., 2021
	*Santolina impressa* (leaves, inflorescence)	Chlorogenic acid, Cynarin	Distilled water, 10 mL/g, 10 min, and 100 °C	Rodrigues et al., 2020
	*Actinidia deliciosa* (kiwifruit)	Quinic acid, Caffeic acid, Caffeoyl hexoside	Distilled water, 50 mL/g, 5 min at 100 °C	Silva et al., 2019
	*A. arguta* (kiwiberry)	Quinic acid, Cis-caftaric acid, Myristin, Caffeoyl hexoside, Luteolin glucuronide, etc		
	*Cymbopogon citratus* (lemongrass) leaves	Caffeic acid, Syringic acid, Citral, Geraniol	Distilled water, 93.8 °C, 11.3 min, and 1:5	Muala et al., 2021
	*Thymus atlanticus* (Moroccan thyme)	Polyphenols, flavonoids (rosmarinic acid, caffeic acid)	Bidistilled water, 25 mL/g, 100 °C, and 30 min	Hmidani et al., 2019
	*Uncaria rhynchophylla*	Uncarophyllofolic acids	Water, ~ 9.2 mL/g, and 30 min	Wang et al., 2019

### Advanced methods of extraction

#### Ultrasound-assisted extraction (UAE)

UAE is performed on both laboratory scale and industrial scale. It is carried out using ultrasonic waves to cause cavitation which leads to the implosion of bubbles in the medium. Cavitation induces collisions, macroturbulence, and disruption of the solid particles. It creates pores and enlarges them which in turn increase the mass transfer rate and the penetration of solvents into the biomass (Gonzalez et al. 2020). UAE can be set up in different configurations depending on the requirements of the extraction process. The transducer is directly dipped in the bulk. It makes the process effective but increases the chances of contamination (Esclapez et al. 2011). Consecutively, different methods of extraction are used to determine the quantity of the desired contents. UAE can be performed with a variety of solvents such as water, ethanol, methanol, acetone, and ethyl acetate, but it must be carried out at a lower temperature to maintain the integrity of thermosensitive compounds (Roohinejad et al. 2017). A few compounds that are extracted using UAE along with sources, solvents, and process conditions are listed in Table 4.

**Table 4:** Principle, sources, compounds, and process conditions employed in ultrasound-assisted extraction

Principle	Sources	Compounds extracted	Process conditions	References
Cavitation induces collisions and shear in the reaction mixture which leads to disruption of solid particles	Banana bract	Dietary fiber	NaOH, 20 kHz, 10 min	Kumar et al., 2021
	Grape seed	Malic acid and Tartaric acid	Water and methanol, 24 kHz, 5 and 10 min	
	Bitter gourd	Total polyphenols or GAE (gallic acid equivalents)	Water, 68.4 °C, 11.6 min, vegetable to water ratio: 0.3 g/L	Chakraborty et al. 2020
	Tannat grape pomace	Polyphenols and Total anthocyanins	Ethanol, 1:20, 30 °C	González et al. 2020
	Saffron (*Crocus sativus*) petals	Crocin,Ssafranal, Catechin, and Epicatechin	Distilled water and NaCl, 40.61 min, power: 135.3 W, 5 g of dried petals added into 100 mL of distilled water containing 0.3 g NaCl	Hashemi et al. 2020
		Delphinidin 3,5-di-O-glucoside		
		Quercitin 3-O-glucoside,		
	*Stevia rebaudiana* Bertoni leaves	Steviol glycosides, Phenolic compounds	Water, 100 °C, 24 h	Žlabur et al., 2015
	*Agaricus bisporus L*	Ergosterol, Gallic acid	Ethanol, 15 min, 375 W	Heleno et al., 2016
	*Anthemis cotula L.* (stinking chamomile)	Anthecotuloid, Caffeoyl quinic acid and Quercetin	96% ethanol, 1:20, 60 min, 30 °C	Sut et al. 2019
	*Morinda citrifolia L.* fruits	Noni polysaccharides	Distilled Water, 1:33, 78 °C, 81.7 min	Li et al. 2020

#### Microwave-assisted extraction (MAE)

This is a technique that assists extraction by irradiating microwaves (frequency range: 300 MHz to 300 GHz) onto the sample (Rehman et al. 2020). Energy associated with these microwaves is converted to thermal energy as the moisture present within the cells starts to evaporate due to ionic conduction as well as dipole rotation, two phenomena associated with microwave technology (Zghaibi et al. 2019). As a result, the cells experience a pressure build-up, eventually causing them to rupture, thus releasing the bioactive compounds (Pinzon et al. 2020). This technique has gained a lot of interest due to its ability to use extraordinarily little quantities of solvents and rapid extraction times, as well as greater reproducibility and control of process conditions such as temperature and pressure (Milani et al. 2020). The principles involved in this technique also facilitate a homogeneous temperature distribution, which also aids in higher yields and favorable heat and mass transfer from the sample to solvent (Pinzon et al. 2020). Table 5 provides an overview of the sources and various compounds extracted using this technique, along with the necessary parameters required for optimal yields.

**Table 5:** Various bioactive compounds extracted by microwave-assisted extraction, along with the principle, respective sources, and process parameters

Principle	Sources	Compounds extracted	Process conditions	References
Microwave irradiation, intracellular moisture evaporation, pressure build-up, and rupture of cells	*Anthemis cotula L.* (stinking chamomile)	Anthecotuloid, Caffeoylquinic acid, and Quercetin	96% ethanol, 1:20, 30 min, 600 W	Sut et al. 2019
	*Miscanthus sinensis* (runo) stem	Runo dye	50% ethanol, 15 s, 540 W	Pinzon et al. 2020
	*Phyllostachys pubescens* (bamboo)	Polyphenols, Favonoids	Methanol, 6.25 g/mL, 105 °C, and 4 min	Milani et al. 2020
	*Nannochloropsis sp.* (microalgae)	Lipids (PUFAs and omega-3 fatty acids)	10% brine 1:20, 100 °C, and 30 min	Zghaibi et al. 2019
	Hemp nut	Cannabinoids (cannabidiol, cannabinol, tetrahydrocannabinol)	Methanol, 375 W, 109 °C, and 30 min	Chang et al. 2017
	*A. nodosum*	Fucose sulfated polysaccharides	1000 W, and 5 min	Garcia-Vaquero et al. 2020
	*Fucus vesiculosus*	Fucose sulfated polysaccharides	Water, 120 psi, 1 min, and 1:25	Rodriguez-Jasso et al. 2011
	*Mangifera indica L.* (mango) peel	Mangiferin	Deep eutectic mixture of lactic acid, sodium acetate, and water (3:1:4), 436.45 W, 19.6 min, and 59.8 mL/g	Pal and Jadeja 2020
	Coriander	Heneicos-1-ene	Ionic solvents (BMIM-BF4) (0.1 M), 800 W, 90 °C, 2 min, 1:10	Priyadarshi et al. 2020

#### Pressurized liquid extraction (PLE)

PLE involves the use of solvents at elevated temperatures, lower than their respective critical points to maintain them in the liquid state. This process exploits the mass transfer properties at elevated temperatures and pressures (Zakaria et al. 2020). The process involves moistening the sample with the solvent. The desired compound desorbs from the sample and gets absorbed in the extraction solvent. The temperature being the key parameter of PLE is used to modify the physicochemical properties of the solvent (Anticona et al. 2020). There are two types of setups used for PLE: static and dynamic, as well as a combination of both. The dynamic system includes a continuous pumping of aliquots of the solvent, the rate being around 0.5–2.5 ml/min. In the static method, the extracted solvent is collected every 5–10 min (Vazquez-Roig and Picó 2015). PLE is used in the contamination analysis in complex matrices such as food. It can be used to identify tenacious organic pollutants (Ridgway 2012). Table 6 lists out a few compounds that are extracted using PLE as well as the sources, required solvents, and process conditions.

**Table 6:** Principle, sources, compounds, and process conditions used in pressurized liquid extraction

Principle	Sources	Compounds extracted	Process conditions	Reference
Extract targeted analytes from a sample matrix into a small amount of S using high Ts and pressures	*Rosmarinus officinalis L.* (rosemary) leaves	Rosmarinic acid, Carnosic acid, Carnosol	183 °C, 130 bar, and 3 min	Hirondart et al. 2020
	*Silybum marianum L. Gaertner* fruits	Silymarin	Acetone, 125 °C, 10 min, and 60 bar	Wianowska and Wiśniewski 2015
	Feijoa leaf	Gallic acid, Catechin and Isoquercetin	Ethanol–water, 80 °C	Santos et al. 2021
	Orange peel	Hesperidin, Naringin, Narirutin, tangeretin, naringenin, hesperidin	75% ethanol, 65 °C, 40 min, and 10 MPa	Anticona et al. 2020
	underutilized chia seeds	Omega 3-rich oils (ALA and Linoleic acid)	Ethanol, 60 °C, and 10 min	Villanueva-Bermejo, 2019
	*Moringa oleifera* leaves	Phenolic compounds	35% ethanol, 128 °C, 20 min	Rodríguez-Pérez et al. 2016
	*Neochloris oleoabundans*	Carotenoids	Ethanol, 100 °C, 20 min, 1500 psi, 0.6 g algae + 2 g sea-sand	Castro-Puyana et al. 2017
	*Fucus vesiculosus*	Gallic, Protocatechuic, and Gentisic acids	58.65% ethanol, 137.18 °C, and 4.68 min,	Sumampouw et al. 2021
	*Chlorella* sp. microalgae	Phenolic compounds	Water, 100 °C and 250 °C, and 5 to 20 min	Zakaria et al. 2020

#### Enzyme-assisted extraction (EAE)

Enzyme-assisted extraction is useful when it comes to the extraction of phytochemicals associated with the cell wall. The presence of cellulose, hemicellulose, and lignin in higher concentrations makes it difficult to implement other popular extraction techniques (Nadar et al. 2018). EAE overcomes this problem by employing enzymes such as cellulase, pectinase, and alpha-amylase involved in the digestion of the cell wall (Azmir et al. 2013). This method has several advantages in terms of environmentally friendly methods and lower consumption of energy and equipment compared to other techniques, reduced usage of toxic solvents, and efficient extraction of thermally sensitive and volatile compounds used as fragrances, flavors, pigments, etc. (Nadar et al. 2018). This method has immense potential and several bioactive compounds of industrial and pharmaceutical importance have been successfully extracted (Table 7) However, enzymes are too expensive to be utilized for extraction of large volumes of substances. Designing efficient ways to scale up such processes is also a challenge (Franco et al. 2020).

**Table 7:** Principle and various sources, bioactive compounds extracted, corresponding enzymes utilized, and optimum process conditions involved in enzyme assisted extraction

Principle	Sources	Compounds extracted	Process conditions	References
Cell-wall digesting enzymes	*C. annuum baydgi*	Capsaicinoids and Carotenoids	Enzymes: *R. nigricans* enzymatic extract (cellulase, hemicellulase, pectinase), 30–36 °C. Concentration: 113.039 µg/mL, 70 min for carotenoids and 45 min for capsaicinoids	Salgado-Roman et al. 2008
	*Chondrus crispus* and *Codium fragile*	Neutral sugars, Uronic acid, Proteins and Sulfates	Enzymes: Cellulase, beta-glucanase, Ultraflo, Neutrase (a protease) (0.5%), 50 °C (water bath), 3 h followed by enzyme denaturing (90 °C, 15 min)	Kulshreshtha et al. 2015
	Waste *Punica granatum L.* (pomegranate) seeds	Oils (PUFAs, punicic acid), Proteins, Insoluble fibers (Cellulose, Hemicellulose, Lignin)	Enzymes: Protease, 45 °C, Concentration: 50 U/g, 14 h, pH 7.2	Talekar et al. 2018
	*Fucus distichus*, *Saccharina latissima* (brown macroalgae)	Fucoidans	Enzymes: Cellic®CTec2 (commercial cellulase), alginate lyase (*Sphingomonas*), 40 °C, pH: 6	Nguyen et at. 2020
	*Helianthus annuus L.* (sunflower) wastes (petals, florets)	Carotenoids (lutein, zeaxanthin, Antheraxanthin, violaxanthin)	Enzyme: Viscozyme® (a multi-enzyme complex) + d,l-menthol/ d,l-lactic acid (2:1), 40 °C, 2 h	Ricarte et al. 2020
	*Scutellaria baicalensis* Georgi	Baicalin	Enzyme: HG-5 enzyme from *Bacillus* spp.	Ma et al. 2021
	*Mentha arvensis L.* (Japanese peppermint)	Essential oils	Enzyme: Cellulase T + hemicellulase 90. Concentration: 2 wt% (both enzymes), 3 h	Shimotori et al. 2020
	*Haematococcus pluvialis*	Astaxanthin	Enzyme: Cellulase (100 U/g) in a 0.2 mol/L sodium acetate buffer, 40 °C. Concentration: 1.5% (w/w), 3 h, pH: 5	Zhao et al. 2019
			Enzyme: Pectinase (7 U/g) in a 0.2 mol/L sodium acetate buffer, 50 °C. Concentration: 0.08% (w/w), 2.5 h, pH: 5,	
	Mushrooms—*Lentinus edodes, Agrocybe aegerita, Pleurotus ostreatus, Agaricus bisporus*	Umami (mainly Monosodium glutamate)	Enzyme: Flavourzyme® + beta-glucanase, S: water, 50 °C. Concentration: 5% v/w, 1 h, pH: 7	Poojary et al. 2017

#### Pulse electric field-assisted extraction (PEFAE)

This extraction technique works on the principle of electroporation. This occurs when cells are exposed to high-intensity electric field pulses that charge cell membranes, eventually creating pores due to increased repulsive forces between membrane constituents, usually after the transmembrane potential crosses a value of 1 V (Gorte et al. 2020). Static bench-scale equipment usually consists of a high voltage power generator, a digital oscilloscope (to monitor voltage, current, frequency, etc.) and a treatment chamber, where the sample is placed (Bozinou et al. 2019). This method is an alternative to other techniques because it increases permeability (and hence, extraction rates) without creating cellular debris, thus increasing the purity of the product (Martínez et al. 2020). In addition to its scalability and selectivity, this method is convenient for the extraction of compounds from wet biomass since it eliminates the need for drying (Carullo et al. 2020). Various compounds that have been previously extracted with this method, along with solvents, sources, and other process parameters, have been listed in Table 8. PEFAE allows exposure of electric pulses without a drastic temperature increase, which prevents the disintegration of thermally unstable compounds (Kokkali et al. 2020). However, process parameters such as field strength and pulse number specifically depend on the composition of the medium and its tendency to generate a potential in response to electric field pulses. High equipment costs limit extensive use of this technology.

**Table 8:** Principle, along with various sources, bioactive compounds extracted, solvents used, and optimum process conditions of pulse electric field-assisted extraction

Principle	Sources	Compounds extracted	Process conditions	Reference
Electroporation	*Arthrospira platensis*	Water-soluble proteins (WSP), C-phycocyanin	Water (aqueous microalgae suspensions 2% w/w, monopolar pulses, 20 kV/cm, and 100 kJ/kg suspension at room temperature	Carullo et al. 2020
	*Saitozyma podzolica* (yeast)	Lipids	Ethanol-hexane-water (18: 7.3: 1), Cell concentration: 20 g/L, electric field: 40 kV/cm, energy: 150 kJ/L suspensions, pulse duration: 1 µsec	Gorte et al. 2020
	*Moringa oleifera* dry leaves	Phenol and Antioxidants	20 mL of double distilled water per gram of ground leaves, 40 min, pulse duration: 20 ms, pulse interval: 100 µsec; field strength: 7 kV/cm	Bozinou et al. 2019
	*Acanthophyllum squarrosum* roots	Saponins	Electric field: 6.4 kV/cm; pulse number: 80	Shahi et al. 2021
	*Oryza sativa* (brown rice)	Gamma oryzanol, Tocopherols, several polyphenols, and fatty acids	Acetone (40%), electric field: 2 kV/cm, S concentration: 5 mL/g, pulse duration: 100 µsec, frequency: 5 Hz	Quagliariello et al. 2016
	*Annona squamosa* (custard apple) leaves	Purpureacin 2, Rutin	Ethanol (70%), electric field: 6 kV/cm, pulse number: 300, energy: 142 kJ/kg, 5 min	Shiekh et al. 2021
	Sea bream and sea bass residues (gills, head, bones)	PUFAs (docosahexaenoic acid, omega-3, etc.), minerals (Ca, P, etc.), Amino acids (arginine, Leucine,Llysine)	Distilled water, 1 mL/mg solids, pulse width: 20 µsec, frequency: 10 Hz, pulse number: 100, electric field: 7 kV	Franco et al. 2020
	*T. chuii* and *P. tricornutum*	Carotenoids, Chlorophyll A, Chlorophyll B	Energy: 100 kJ/kg, pulse duration: 100 ms, pulse frequency: 2 Hz	Kokkali et al. 2020
	*Tetraselmis chuii*	Carotenoids	24-h extraction using DMSO, subsequent PEFAE-electric field: 1 kV/cm, pulse number: 400	
		Chlorophyll B	Water, electric field: 3 kV/cm, pulse number: 45; extraction time: 24 h	
	*Phaeodactylum tricornutum*	Carotenoids, Chlorophyll A	DMSO (50%), electric field: 3 kV/cm, pulse number: 45; extraction time: 4 h	

#### Supercritical fluid extraction (SFE)

SFE is a highly specific extraction method used in pharmaceutical, chemical, and food industries, to extract surfactants, food additives, and fragrant compounds, with CO_2_ as the main solvent. Thermosensitive bioactive compounds face minimal damage because of the low critical temperature of CO_2_ (Uwineza and Waśkiewicz 2020). Table 9 lists out a few compounds extracted with SFE along with the sources, solvents, and process conditions. SFE operates in supercritical solvent conditions at elevated temperatures and pressures which makes the process more efficient as it enables the gas-like solvent to penetrate the solid matrices. The key conditions: temperature and pressure of the supercritical fluid can significantly alter the selectivity as well as solubility (Villanueva-Bermejo et al. 2019). Supercritical CO_2_ is the most common solvent used for SFE as it exists as a gas at room temperature and can be separated from the extraction mixture easily. Furthermore, it has moderate critical temperature and pressure (Al Jitan et al. 2018). However, the low polarity of CO_2_ makes it undesirable to extract polar compounds. To overcome this challenge, NO_2_ is used as an alternative to extract polar compounds (Capuzzo et al. 2013). An SFE setup involves a condenser, an extractor, and separators. The CO_2_ from the condenser is pressurized and passed into the system whilst the temperature is being regulated. The extractor is depressurized, and the cylinder containing the raw material is fed into the extractor. The system is pressurized, and the scCO_2_ is allowed to enter the extractor in a regulated flow till the extraction process is complete. The scCO_2_ is recirculated to the condenser, and the extracts will be collected in the separators (Baysal et al. 2000). Costly setup is the major disadvantage of SFE (Uwineza and Waśkiewicz 2020).

## Characterization of bioactive compounds

A plethora of bioactive compounds exist in multi-component states which make the isolation and separation and characterization process a crucial task. Characterization is a process that is performed to obtain a pure form of the target bioactive compound which helps in determining the amount, structure, and biological activity of the compound (Mahato et al. 2019). It plays a key role in the identification of potentially bioactive compounds with novel functionalities, including but not limited to drugs and antimicrobials. This is important in areas where these substances were traditionally used, but their exact chemical structure and properties were left undocumented (Ayalew 2020). Various chromatographic techniques have been developed to fractionate various kinds of compounds present in a single extract, such as ion exchange chromatography (IEC), thin layer chromatography (TLC), size exclusion chromatography (SEC), high-speed counter current chromatography (HSCCC), and high-performance thin layer chromatography (HPTLC). More advanced methods such as nuclear magnetic resonance (NMR), mass spectrometry (MS), Fourier transform infrared spectroscopy (FTIR), and so on are more selective and enable an analysis of bioactive compounds at molecular levels (García-Vaquero and Rajauria 2018). Table 10 describes various methods of characterization that are employed, in addition to the principles involved and compounds that have been identified by each method.

## Applications of bioactive compounds

**Fig. 2:**
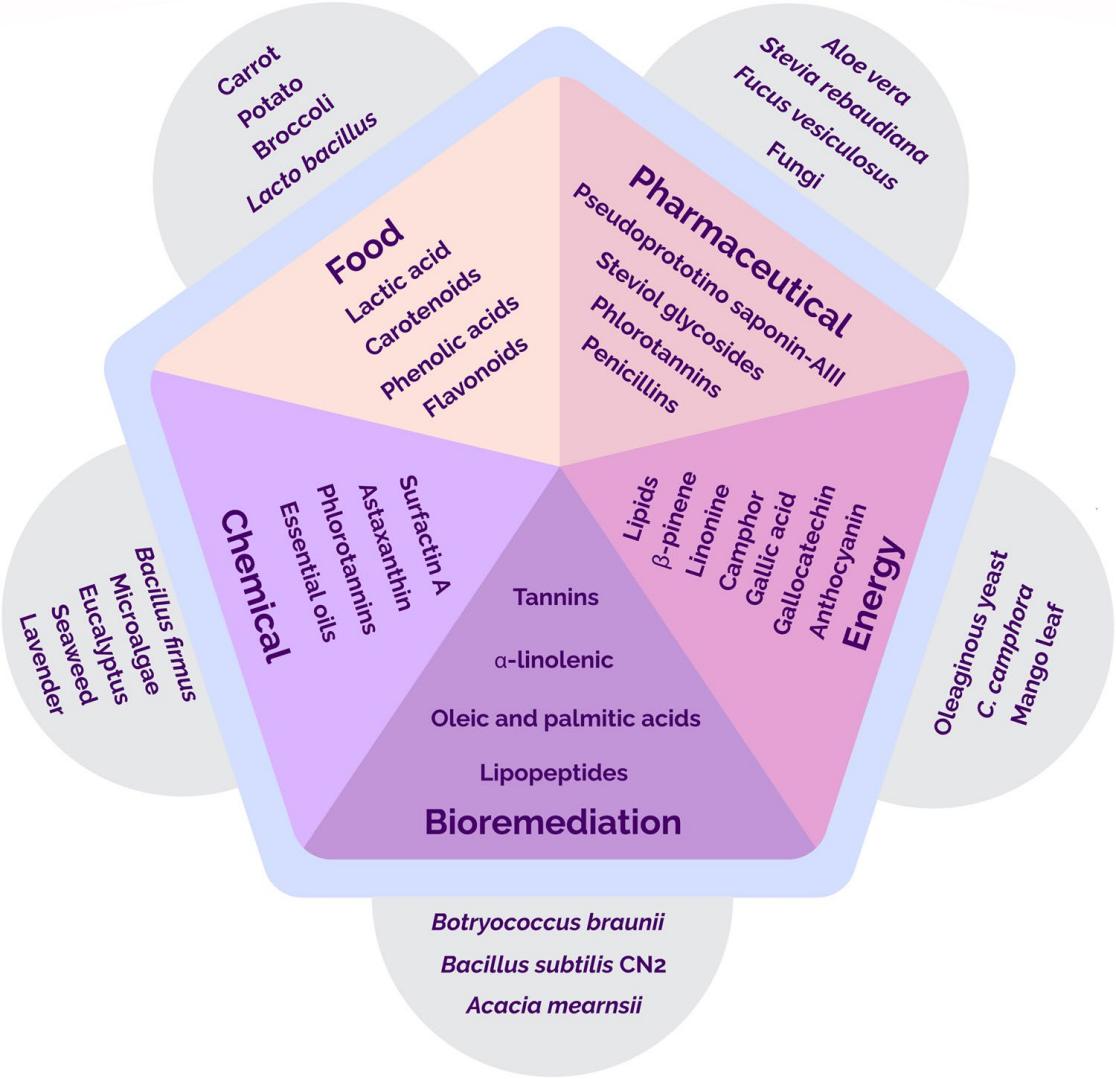
Five major applications of bioactive compounds, along with a few sources as well as important compounds

In this study, the applications of bioactive compounds in five major sectors such as food, pharmaceutical, bioremediation, energy, and chemical along with their major sources and important compounds extracted were discussed (Fig. 2).

### Food sector

Bioactive compounds add a substantial value to the food industry. Food and nutrient supplements (Talekar et al. 2018), food coloring (Chhikara et al. 2019), meat and meat products (Pogorzelska-Nowicka et al. 2018), etc. all contain bioactive compounds necessary for the human body as mentioned before. They are added as a food enhancer as well, for example, carotenoids, curcumin, and anthocyanins are used as coloring agents; ascorbic acid is used widely as an additive to prevent oxidation in foods; vanillin and cinnamaldehyde are used as flavoring agents. Fermentation is one of the main areas under the food industry that produces a lot of bioactive compounds (Sadh et al. 2018) such as single-cell proteins used as an alternative source for protein (Ritala et al. 2017); lactic acid used for acidulation and preservation (Miller et al. 2011); xanthan used as an emulsifier, thickener, and stabilizer (Habibi and Khosravi-Darani 2017); laccase used for baking and in the beverage industry as a stabilizer (Mayolo-Deloisa et al. 2020); astaxanthin used as a coloring agent (Gwaltney-Brant 2021); citric acid used as a food preservative and flavoring agent (Kazmi and Clark 2012); fumaric acid used as an acidulant (Karaffa and Kubicek 2021); and others. The main sources of such bioactive compounds are fruits, wastes produced in the wine industry, plant, fruit, and vegetable waste like peels, seeds, and pomace (Shirahigue and Antonini 2020). Common fruits such as apple, mango, plum, banana, and citrus fruits contain phenolic acids, flavanols, carotenoids, anthocyanins, and lipids. Common vegetables like potato, carrot, beetroot, and broccoli contain carbohydrates, phenolic acids, carotenoids, and flavonoids. A few other sources of carotenoids are *Neochloris oleoabundans*, *C. annuum baydgi*, *Helianthus annuus L.* (sunflower) wastes (petals, florets), *T. chuii*, and *P. tricornutum*. Chokeberry fruit, papery skin of *Allium cepa L.* var. *Ascalonicum*, strawberry, raspberry, blueberry, blackberry, and Tannat grape pomace are a few sources of anthocyanins. Phenols and polyphenols are extracted from *Thymus atlanticus* (Moroccan thyme), bitter gourd, Tannat grape pomace, *Phyllostachys pubescens* (bamboo), and *Oryza sativa* (brown rice) to name a few sources. The sources mentioned above are listed in the extraction tables (Tables 1–9).

**Table 9:** Principle, sources, compounds extracted, and process conditions used in supercritical fluid extraction

Principle	Sources	Compounds extracted	Process conditions	References
Separating extract from the matrix using supercritical fluids like scCO_2_	*Mentha spicata*	Flavonoids (luteolin)	scCO_2_, pressure: 200 bar, 60 °C, and 60 min	Puri et al. 2012
	*Feijoa* leaf	Gallic acid, catechin, and isoquercetin	scCO_2_, 15% ethanol–water (as cosolvent), 210 min, pressure: 30 MPa, and 55 °C	Santos et al.. 2021
	Underutilized Chia seeds	Omega 3-rich oils (ALA and linoleic acid)	scCO_2_, pressure: 45 MPa, 60 °C, 240 min, and 40 g/min	Villanueva-Bermejo 2019
	Leaves of *piper amalago*	Pyrrolidine	scCO_2_, Co-solvents: ethanol, methanol, and propylene glycol 5% (v/v), pressure: 150, 200, and 250 bar, 40, 50, and 60 °C, 20, 40, and 60 min, CO_2_ flow rate: 3 mL/min, and particle size: 0.757 mm	Uwineza and Waskiewicz 2020
	*Catharanthus roseus*	Vinblastine and vincristine	scCO_2_, Co-S: ethanol 2, 5 and 10% (v/v), pressure: 300 bar T: 40, 50, and 60 °C	
	*Artemisia annua L*	Artemisinin	scCO_2_, pressure: 100 bar, 40 °C, CO_2_ flow rate: 13.3–20 g/min	
	Dried ivy leaves	Chlorophyll	scCO_2_, Co-S: ethanol (80/20 v/v), 25 °C, pressure: 15 MPa, and 30 min	Lefebvre et al. 2021
	*Haematococcus pluvialis*	Astaxanthin	scCO_2_, 50 °C, pressure: 50 MPa, 175 min, and flow rate: 2 L/min	Álvarez et al. 2020
	*Rana chensinensis* ovum	*Rana chensinensis* ovum oil (eicosapentaenoic acid, α-linolenic acid, docosahexaenoic acid, arachidonic acid, linoleic acid, and oleic acid)	scCO_2_, pressure: 29 MPa, flow: 82 L/h, 50 °C, and 132 min	Gan et al. 2020

**Table 10:** Various chromatographic and spectrometric methods of characterization, along with their basic principle, sources, and compounds identified or separated

Name	Basic principle	Sources	Compounds identified/separated	Reference
Thin layer chromatography (TLC)				
	The compound having polarity like that of the solvent will get adsorbed faster than other compounds			Santiago 2013
		Citrus fruits	Tangeretin, 5′-demethyltangeretin, nobiletin, 3′-demethylnobiletin, 4′-demethylnobiletin, 3′4′-demethylnobiletin, 5-demethylnobiletin, 5,3′-demethylnobiletin, 5,4′-demethylnobiletin, 5,3′,4′-demethylnobiletin, naringenin, hesperetin	Li et al. 2020
		*Streptomyces misionensis V16R3Y1* Bacteria extracts	Hexahydro-3-(2-methylpropyl) pyrrolo [1,2-a] pyrazine-1, 4-dione followed by N-valeryl-l-proline decyl ester, benzene, acetamide, 2-(ethylhexyl)-hexylsulfate,5-isopropylidene-3,3-dimethyl-dihydrofuran-2-one	Saadouli et al. 2020
Ion exchange chromatography (IEC)				
	Separation of ionized molecules based on their charge. Exchangers having positive/negative charged species retain unlike charges in a column, allowing like charges to pass through			Masoodi et al. 2021
		Brown algae	Fucoidans (fucose, uronic acids, galacturonic acid, glucuronic acid, sulfates)	Sichert et al. 2021
		*Crotalus durissus terrificus* venom	Bordonein L. (L. amino acid oxidase)	El-Aziz et al. 2020a, b
		*Walterinnesia aegyptia* (Egyptian black snake)	Walterospermin	El-Aziz et al. 2020a, b
Size exclusion chromatography (SEC)				
	Molecules in the extract are separated according to their sizes (molecular weights)			Mahato et al., 2019
		*Bothrops atrox* venom	Batroxase	El-Aziz et al. 2020a, b
		Hazelnut and walnut shells	Triglycerides, fatty acids, steryl esters, monosaccharides, phenols	Herrera et al., 2020
		*Euglena cantabrica*	Paramylon, glycans	Muñoz-Almagro et al., 2020
High-speed counter-current chromatography (HSCCC)				
	Fully liquid phase chromatographic technique. The separation is achieved without using any solid phase. The stationary liquid phase is retained on the column by gravitational or centrifugal forces alone			Garcia-Vaquero and Rajauria 2018
		*Polygonum multiflorum* roots	Gallic acid, Catechin, Epicatechin, Polydatin, Piceatannol, Rutin, Resveratrol, Isorhapontigenin, Hyperoside, Rhein, Emodin, 2,3,5,4′-Tetrahydroxy stilbene-2-Ο-β-D-glucoside	Liu et al. 2020
		*Lycium barbarum* fruits	Zeaxanthin, Zeaxanthin monopalmitate, Zeaxanthin dipalmitate	Gong et al. 2020
		*Dipsacus asper* roots	Iridoid glycosides, Triterpenoid saponins	Yu et al. 2020
High-performance thin layer chromatography (HPTLC)				
	The principle is like TLC but with different particle size distribution and thickness of sorbent layers			Maimaiti et al. 2020
High-performance thin-layer chromatography-heated electrospray ionization-high-resolution mass spectra (HPTLC-HESI-HRMS)		*Abelmoschus* *moschatus*	Behenic acid, Arachidic acid, Stearic acid, Oleic acid, Linoleic acid, Palmitic acid	Chandana and Morlock 2021
		*Vernonia anthelmintica*	3,4-O-dicaffeoyl Quinicacid, 3,5-O-dicaffeoyl quinic acid, 4,5-O-dicaffeoyl quinic acid	Maimaiti et al. 2020
		*Musa acuminata* peel	Quercetin, Catechin, Rutin	Vijay et al. 2019
Nuclear magnetic resonance (NMR)				
	An atom placed under a strong magnetic field will respond with its nuclear spin at a certain frequency			Gjuroski et al. 2021
NMR with mass spectroscopy		*Pseudomonas aeruginosa UWI-1*	Tris(1H-indol-3-yl) methylium, bis(indol-3-yl) phenylmethane, indolo (2, 1b) quinazoline-6, 12 dione	Ramkissoon et al. 2020
		*Solidago gigantea* *Ait.* root extract	Kingidiol, Epoxy-hemiacetal, Clerodane lactone (hautriwaic lactone), Solidagoic acid A, Solidagoic acid B	Móricz et al. 2021
(1D and 2D NMR)		*Ardisia elliptica*	Quercetin, Kaempferol, Myricetin derivatives, α-amyrin, β-amyrin, squalene oxide, Ardisianoside, Friedelane derivatives, Ardisenone, Ardisiphenol B, Ardisinol II, Ardisianone derivatives, Embelin, Gallic acid	Wong et al. 2021
Fourier transform infrared spectroscopy (FTIR)				
	Generates a sample-specific FTIR spectrum representing the composition of various molecules depending on the extent of infrared radiation absorbed			Vogt et al. 2019
		*Lantana camara* leaf oil	Alcohols, Carboxylic acids, Alkanes, Ketones, Primary amines, Phenols	Ayalew 2020
		*Glycosmis pentaphylla*	Carbonyl, Amide, Imines, Phenyl ether, Furan groups	Murugan et al. 2020
		Microalgae (*S. platensis*)	Methylene (carotenoids), Carbonyl (phytosterols), Flavone phenyl ring, Ketones (flavonoids), Aromatic groups, Phenyl ether linkages	Lopez-Hernandez et al. 2020
Mass spectrometry (MS)				
	Measuring charge to mass ratio of ionized molecules			Alsenani et al. 2020
Along with UHPLC-Q-TOF–MS (the ultra-high performance liquid chromatography-quadrupole time-of-flight mass spectrometry)		*I. galbana*	Pheophytin A, Trilinolenic glyceride	
		*Scenedesmus* sp. NT8c	A-Linolenol, Stearic acid, Hexadecanoic acid	
		*Chlorella* sp. FN1	Pheophytin A, Ester	
Paper spray mass spectrometry PS ( −)-MS	Negative ionization	*Eriobotrya japonica* Lindl. leaves	Malic acid, 2-Hydroxy-3-(2-hydroxyphenyl), Propanoic acid, Trihydroxy-octadecadienoic acid, Caffeic acid, Quinic acid, 5-p, Coumaroylquinic acid, Chlorogenic acid, 5-Feruloylquinic acid, Catechin, Hexose Feruloylglycoside, Kaempferol-xylose, Kaempferol-rhamnoside, Naringenin hexoside, Kaempferol-glucoside, Quercetin-3-O-glucoside, Taxifolin hexoside, Caffeoyl derivative hexose, Kaempferol-hexose malic acid, Procyanidins B2, Kaempferol-3-O-rutinoside, Rutin, Ursolic acid/Oleanolic acid	Silva et al. 2020

### Pharmaceutical and therapeutic sector

The development of completely novel compounds for therapeutics is a daunting and time-consuming task (Sinha and Häder 2021). This has helped steer major advances in the utilization of a vast diversity of bioactive compounds already found in nature, as well as disciplines like ethnopharmacology, involved in the systematic research and exploration of sources that have traditionally been used as medicine (Suntar 2020), including higher plants (Silva et al. 2019), microalgae, seaweeds (Rodriguez-Jasso et al. 2011), microorganisms (Ramkissoon et al. 2020), fungi (Heleno et al. 2016), and marine organisms (Franco et al. 2020). Plants such as *Aloe vera*, consisting of pseudoprototinosaponin-AIII and prototinosaponin-AIII (Shrinet et al. 2021), alkaloids, triterpenes, thiocyanates, cardiac glycosides, and cyanogenic glycosides, among others, extracted from *Terminalia catappa* (Behl and Kotwani 2017) as well as Steviol glycosides in *Stevia rebaudiana* leaves (Zlabur et al. 2015), have antidiabetic properties. Baicalein, a flavone obtained from the dried roots of *S. baicalensis Georgi*, is known for its anti-cancer and anti-inflammatory activities and has been used to treat several gastrointestinal ailments such as gastric ulceration, liver fibrosis, and so on (Xie et al. 2019). Comparable properties have been observed in silymarin (treatment of liver disorders as well as antitumor activity), extracted from *Silybum marianum L. Gaertner* (Wianowska and Wiśniewski 2015). The extracts of *Anthemis cotula L.* (stinking chamomile) were found to have potential in the treatment of Alzheimer’s disease and skin hyperpigmentation conditions (Sut et al. 2019). Nutraceuticals such as quercetin and kaempferol have also been employed for managing similar neurodegenerative disorders (Makkar et al. 2020).

Other extremely important sources of bioactive compounds include algae as well as marine organisms. Fucose-sulfated polysaccharides, extracted from brown algae such as *A. nodosum* and *Fucus vesiculosus*, have been proven to be beneficial antioxidants and anticoagulants (Garcia-Vaquero et al. 2020), in addition to being anti-inflammatory and antiviral (Rodriguez-Jasso et al. 2011). *Fucus vesiculosus* is also rich in phlorotannins, used in the treatment of goiter, obesity, rheumatoid arthritis, asthma, etc. (Catarino et al. 2019). The extracts of *Chondrus crispus* and *Codium fragile* have successfully exhibited activity against the *Herpes simplex* virus (HSV) (Kulshreshtha et al. 2015).

Research on bacterial and fungal bioactive compounds has explored antimicrobial and anti-cancer properties (Sinha and Hader 2021). Fungi are sources of the very first antibiotics, such as penicillins, carbapenems, and cephalosporins. Compactin and lovastatin have been very instrumental as cholesterol-lowering agents (Hoeksma et al. 2019). Bioactives obtained from fungal sources were found to exhibit antibacterial, antiviral, anti-cancer properties in addition to being immunostimulants (Poojary et al. 2017). Several indole alkaloid compounds have been extracted from bacteria such as *Pseudomonas aeruginosa* UWI-1 having antibiotic potential (Ramkissoon et al. 2020). Scleritodermin A, a compound isolated from *Scleritoderma nodosum*, was found to be effective in the treatment of human colon, breast, and ovarian tumors (Sinha and Hader 2021).

Various bioactive compounds of pharmaceutical importance have been extracted from animal sources as well. Franco et al. (2020) suggested using residues of sea bream and sea bass (gills, head, bones) to extract high-value antioxidants. *Rana chensinensis* ovum oil was found to have beneficial unsaturated fatty acids, instrumental in the prevention of cardiovascular as well as cerebrovascular diseases (Gan et al. 2020). Advances in venomics have helped in the extraction and isolation of animal (snake) venom, useful in the drug discovery and development of antivenom (El-Aziz et al. 2020a, b). A range of sources along with relevant compounds, many of which are of pharmaceutical interest, have already been listed in Tables 1–10.

### Bioremediation sector

Bioactive compounds have found a range of applications in bioremediation sectors as well, in the form of coagulants (Ibrahim et al. 2021), biofilms (Mugge et al. 2021), bioactive extracts (Zerrifi et al. 2018), and so on. A wide range of bioactive compounds obtained from various sources has shown promising activity against harmful algal blooms (HAB), including α-linolenic, oleic, and palmitic acids from *Botryococcus braunii*, diethyl phthalate from *Stoechospermum marginatum*, and so on (Zerrifi et al. 2018, 2021). Similarly, bioaccumulation of nutrients, as well as metals (Cd, Cu, Zn, Pb, Cr) by *Sargassum*, has triple benefits in terms of reducing eutrophication and coastal metal pollution in addition to sequestering metals and useful bioactive compounds which could then be used in pharmaceutical, cosmetic, food, and fertilizer industries (Saldarriaga-Hernandez et al. 2020). d’Errico et al. (2020) reported the capability of a fungal endophyte, *Drechslera* (strain 678) to have dual functions as a biopesticide, due to the presence of compounds such as monocerin, as well as for bioremediation of methyl tert-butyl ether, a soil contaminant, usually used as a gasoline additive.

Tannins have also been widely used in wastewater treatment plants. Tannin-based coagulants have been employed to remove turbidity and flocculate suspended solids (Ibrahim et al. 2021). Condensed tannins sourced from *Acacia mearnsii* and tannic acid have been used to remove both cationic and anionic dyes from water (Grenda et al. 2020). Das et al. (2020) have also reviewed the use of tannin cryogels and wattle tannins in the removal of heavy metals and methylene blue, respectively, from contaminated water.

Biofilms formed by marine and intertidal bacteria have a lot of potential in bioremediation. Mugge et al. (2021) studied the changes in bacterial populations and biofilm compositions in surface and deep-sea water when exposed to crude oil or chemical dispersants, which is promising in the management and clean-up of oil spills. Lipopeptides obtained from *Bacillus subtilis CN2* showed interesting properties about the degradation of polycyclic aromatic hydrocarbons and recovery of motor oil from contaminated soil (Bezza and Chirwa 2015). Hence, bioactive compounds are promising when it comes to areas like wastewater treatment and hydrocarbon degradation.

### Energy sector

With the upsurge in human population, renewable energy like biofuels and other sustainable practices has become essential. Five billion tonnes of biomass waste are produced in the food and agroforestry industry. It has ample potential in the production of bioactive compounds which can be utilized as biofuels to reduce biomass waste. Ethanol and vegetable oil are widely produced as biofuels in biorefineries (Ferreira-Santos et al. 2020). Gorte et al. (2020) demonstrated pulse electric field treatment to extract lipids from fresh oleaginous yeast cells. Single-cell oils or microbial oils that are extracted from yeasts, fungi, microalgae, and bacteria can be utilized as alternative fuels, although the extraction process is expensive and not efficient. These remain as the major drawbacks still left to tackle. *C. camphora* is a potential renewable energy source, and the mass production along with the volatile constituents (camphor, eucalyptol, limonene, β-pinene) has been studied (Zhang et al., 2020a, b). Additionally, sugar-based waste (sugar cane, sugar beets), animal waste (cow, swine, poultry), food industry waste, starch-based wastes (corn), lignocellulosic waste (switchgrass, micanthus, corn stover, corn fiber), and glycerine serve as sources of bioenergy and biofuels such as ethanol, methanol, and butanol (Swain 2017). Microbial fuel cells (MFCs) use microbes to generate electricity. They have shown enhanced power density with electron shunting capabilities of a few secondary metabolites such as epigallocatechin-3-gallate, gallic acid, gallocatechin, and anthocyanin. The addition of fungal and algal metabolites in the MFCs improves electricity production (Nath and Ghangrekar 2020). Condensation of β-pinene is processed to form dimers which is an excellent option for a renewable and high energy–density jet fuel and can also be used as diesel (Jung et al. 2016). Effects of a dual biofuel blend consisting of different concentrations of jatropha biodiesel and turpentine oil were studied in a single-cylinder diesel engine and are a cost-effective alternative for fossil fuels. There was a reduction in carbon monoxide, hydrocarbon, and nitrous oxide emissions by 13.04%, 17.5%, and 4.21%, respectively, but an increase in CO_2_ emissions by 11.04% (Dubey and Gupta 2018).

### Chemical sector

Bioactive compounds have applications in various chemical industries, including but not limited to polymers and biomaterials (Nogueira et al. 2020), dyes and textiles (Agnhage et al. 2017), leather processing (Das et al. 2020), perfumes, and cosmetics (Sharmeen et al. 2021). Certain bioactive compounds (oils, fatty acids) have traditionally been used (in the oil and soap industries) (Ng et al. 2021), whereas the toxicity and unsustainability of conventional chemicals, processes, or end-products have recently accelerated the usage of bio-based substitutes in novel materials, catalysts, as and certain raw materials in the industry (Chin et al. 2021; Basak et al. 2021).

Spiridon et al. (2020) developed a biomaterial with cellulose, collagen, and polyurethane as its constituents, to facilitate the controlled release of antioxidants such as tannin and lipoic acid, with a potential for biomedical and cosmetic applications. Similar approaches of encapsulation of antioxidants were carried out using Aloe vera agrowastes, by incorporating them into electro spun nanofibers made from polyethylene oxide (Solaberrieta et al. 2020). These technologies have also enabled the development of biodegradable and even edible food packaging films with enhanced antimicrobial, antioxidant, and mechanical properties, achieved by a combination of biopolymers (starch, chitosan, gluten) and bioactive (essential oils, polyphenols, carotenoids) (Nogueira et al. 2020). Other interesting properties, such as flame retardancy, antibacterial, and UV light protection in textiles have been achieved by using tannin-based macromolecules (Basak et al. 2021). Dyes, such as the run dye from the core stem tissues of *Miscanthus sinensis* Andersson (Pinzon et al. 2020) and naturally sourced anthraquinone dyes from the roots of *Rubia tinctorum L.* (Agnhage et al. 2017), have also been produced.

Various essential oils such as lavender, carvone, linalool, limonene, citronellol, and eucalyptus are popular choices in the perfume and cosmetic industry. In addition to fragrance, they also act as preservatives and active ingredients and have beneficial effects on the skin (Sharmeen et al. 2021). Phlorotannins, polysaccharides (laminarin, carrageenan, etc.), astaxanthin, and several bioactive peptides present in seaweed and microalgae have been reported as excellent sources for cosmeceuticals due to their anti-aging, anti-acne, antimicrobial, skin glow enhancement, moisture retention, UV protection, and anti-allergic properties (Jesumani et al. 2019).

The industrial extraction of oils (palm, coconut, and castor oils) is another important sector of the chemical industry, due to a wide range of applications, ranging from edible oils and hair care products (Ng et al. 2021) to the manufacture of soaps and grease (Patel et al. 2016). Moreover, several petrochemical industries in Malaysia are looking into the possibility of using palm oil as well as glycerol sourced from vegetable oil as feedstocks in the manufacture of lubricants (Chin et al. 2021), shifting resources from fossil fuels to bioactive compounds. Similarly, castor oils are used in the manufacture of biodegradable polyesters as well as lubricants and paints (Patel et al. 2016).

Tannins, on the other hand, are widely abundant and industrially important bioactive compounds that have been used in the leather processing and wood (adhesive and preservation) industry for centuries (Das et al. 2020). In addition to this, several novels and sustainable alternatives to 3D printing (Liao et al. 2020) using tannins have been explored. Bioactive compounds thus have a high potential for creating a sustainable chemical industry if measures are taken for efficient extraction and conservation of biodiversity.

## Limitations

Conventional extraction processes are helpful; however, they are inefficient and time-consuming. To overcome the said limitations, non-conventional extraction methods have been designed. However, they have certain demerits as well. Susceptibility of thermosensitive compounds, non-uniformity of extraction in large-scale industries, and high maintenance costs and CO_2_ consumption leading to high-value compounds are the challenges faced in UAE, MAE, and SFE, respectively. For example, in the case of SFE, astaxanthin and phycobiliproteins in microalgae are high-value compounds due to the expensive extraction and purification processes (Roohinejad et al. 2017). Various other limitations specific to each extraction method have already been discussed.

A major impediment to the widespread use of bioactive compounds is the varying stability and loss of activity, especially in foods, as most experiments verifying beneficial properties are done under controlled conditions. Variation among individuals is also a crucial factor that must be considered while studying the nutritional and therapeutic benefits of bioactive compounds. Differences in processes such as absorption and metabolism, as well as diversity in age, gender, and lifestyles, could result in varied effects of such compounds in a population. From the ecological perspective, meeting the growing demands for bioactive compounds is bound to exert a lot of pressure on biodiversity, land, and marine resources, which could threaten the survival of exceedingly rare species.

Challenges that are frequently overlooked about bio-analytical methods of characterization are interference to a considerable extent, clean-up during sample preparation, low sensitivity, accuracy, and unreliable methods to name a few. Other aspects, such as bioavailability, bio accessibility, safe and “green” production practices, safety, and toxicology, must be considered as well, especially when downstream processes account for 50–80% of the production value (Cuellar-Bermudez et al. 2015).

## Future scope

The advent of non-communicable diseases such as cancer, obesity, diabetes, and so on over the last few decades, the instances of antibiotic resistance in pathogenic microorganisms, the increased awareness for sustainable products, bioremediation efforts, and the recent pandemic have led to a spike in demand for healthier, natural, immune-boosting and bio-fortified foods, novel antibiotics and pharmaceuticals, bio-based raw materials for various processes, and biomaterials with novel functionalities. A report by Grand View Research, Inc. (2016) states that the global market size for bioactive ingredients is expected to cross USD 51.71 billion by 2024, with functional foods and beverages contributing to 25% of the market, sourced from plants and marine organisms (https://www.grandviewresearch.com/press-release/global-bioactive-ingredients-market). This surge in demand necessitates further research into sustainable and effective methods of screening, extraction, characterization, processing, and commercialization of good-quality bioactive compounds. Fu et al. (2019) have suggested a shift to multi-targeted approaches to screening multiple bioactive compounds simultaneously with the help of biosensor and microfluidic chip-based technologies, as opposed to conventional chromatographic methods. The mechanism of action of bioactive compounds in certain cases is best understood during in vivo screening methods; hence, the scope of discovery of these compounds is limited when screening is confined to in vitro assays (Ahamefule et al. 2020). Designing effective in vivo assays, especially for antibiotic activity, could help in screening such compounds utilizing novel mechanisms. Ways to improve selectivity and yield of extraction, such as modeling solvent-compound interactions and affinities, as well as optimization of physical parameters in case of non-conventional methods must be explored further to decrease costs and facilitate scalability.

Metabolic engineering is a potential tool to facilitate microbial production of bioactive compounds such as terpenoids, omega-3 PUFAs, and so on, to tackle the exploitation of rare and limited marine resources for commercial production. Other approaches of finding optimum sources from by-products of food processing industries and agricultural residues can help in implementing sustainable methods of waste recycling and high-value product recovery, boosting a circular economy.

Nanoencapsulation strategies have a good potential when it comes to retaining bioavailability, enhancing stability, and facilitating the controlled release of bioactive compounds while being delivered into functional food items. Encapsulation within naturally assembling structures and biopolymer films in food packaging are a few approaches. The challenge lies in making them economically viable alternatives to conventional solutions.

## Conclusion

This article has reviewed bioactive compounds, their extraction methods, characterization methods, applications, limitations, and future scope, all together. The popular conventional and non-conventional extraction methods to extract bioactive compounds along with tables containing the basic principles, sources, latest compounds extracted, solvent or enzymes used, and process conditions were reviewed and listed. Bio-analytical characterization methods were elucidated with the help of a table. The applications of bioactive compounds in the food, pharmaceutical, bioremediation, energy, and chemical sectors were documented diffusely. Moreover, the limitations and challenges faced in the extraction and characterization processes were compiled. The prospects of the bioactive compounds were put together considering the ongoing pandemic situation although it needs further investigation. This gives an insight into the value of bioactive compounds from the perspective of human health and the sustainability of global resources. As the technology ameliorates, the potential of bioactive compounds in various sectors is bound to escalate, thus unlocking new possibilities.

## Data Availability

The datasets used and/or analyzed during the current study are available from the corresponding author on reasonable request.
